# MAL2 and tumor protein D52 (TPD52) are frequently overexpressed in ovarian carcinoma, but differentially associated with histological subtype and patient outcome

**DOI:** 10.1186/1471-2407-10-497

**Published:** 2010-09-17

**Authors:** Jennifer A Byrne, Sanaz Maleki, Jayne R Hardy, Brian S Gloss, Rajmohan Murali, James P Scurry, Susan Fanayan, Catherine Emmanuel, Neville F Hacker, Robert L Sutherland, Anna deFazio, Philippa M O'Brien

**Affiliations:** 1 Molecular Oncology Laboratory, Children's Cancer Research Unit, The Children's Hospital at Westmead, Westmead, New South Wales, Australia; 2 The University of Sydney Discipline of Paediatrics and Child Health, The Children's Hospital at Westmead, New South Wales, Australia; 3 Cancer Research Program, Garvan Institute of Medical Research, Darlinghurst, Sydney, New South Wales, Australia; 4 Tissue Pathology and Diagnostic Oncology, Royal Prince Alfred Hospital, Camperdown, New South Wales, Australia; 5 Discipline of Pathology, The University of Sydney, Camperdown, New South Wales, Australia; 6 South East Area Laboratory Service, Prince of Wales Hospital, Randwick, New South Wales, Australia; 7 Westmead Institute for Cancer Research, The University of Sydney at Westmead Millennium Institute, Westmead, New South Wales, Australia; 8 Department of Gynaecological Oncology, Westmead Hospital, Westmead, New South Wales, Australia; 9 Gynaecological Cancer Centre, Royal Hospital for Women, Randwick, New South Wales, Australia; 10 School of Women's and Children's Health, University of New South Wales, Randwick, Australia; 11 St Vincent's Clinical School, University of NSW, Sydney, New South Wales, Australia

## Abstract

**Background:**

The four-transmembrane MAL2 protein is frequently overexpressed in breast carcinoma, and MAL2 overexpression is associated with gain of the corresponding locus at chromosome 8q24.12. Independent expression microarray studies predict *MAL2 *overexpression in ovarian carcinoma, but these had remained unconfirmed. MAL2 binds tumor protein D52 (TPD52), which is frequently overexpressed in ovarian carcinoma, but the clinical significance of MAL2 and TPD52 overexpression was unknown.

**Methods:**

Immunohistochemical analyses of MAL2 and TPD52 expression were performed using tissue microarray sections including benign, borderline and malignant epithelial ovarian tumours. Inmmunohistochemical staining intensity and distribution was assessed both visually and digitally.

**Results:**

MAL2 and TPD52 were significantly overexpressed in high-grade serous carcinomas compared with serous borderline tumours. MAL2 expression was highest in serous carcinomas relative to other histological subtypes, whereas TPD52 expression was highest in clear cell carcinomas. MAL2 expression was not related to patient survival, however high-level TPD52 staining was significantly associated with improved overall survival in patients with stage III serous ovarian carcinoma (log-rank test, p < 0.001; n = 124) and was an independent predictor of survival in the overall carcinoma cohort (hazard ratio (HR), 0.498; 95% confidence interval (CI), 0.34-0.728; p < 0.001; n = 221), and in serous carcinomas (HR, 0.440; 95% CI, 0.294-0.658; p < 0.001; n = 182).

**Conclusions:**

MAL2 is frequently overexpressed in ovarian carcinoma, and TPD52 overexpression is a favourable independent prognostic marker of potential value in the management of ovarian carcinoma patients.

## Background

Epithelial ovarian carcinoma is a disease often characterised by poor outcome, despite intensive efforts to improve early disease detection, and to understand the causes of frequent treatment failure [1,2]. To improve our understanding of the underlying molecular basis of this histologically heterogeneous group of tumours, large numbers of cytogenetic and comparative expression studies have been undertaken. Cytogenetic analyses have consistently identified chromosome 8q gain as a common event in ovarian carcinoma [summarised in 3], and in other cancer types [4,5], and recent studies continue to highlight the fact that several distinct regions along chromosome 8q are increased in copy number [6-8]. One such region occurs at chromosome 8q24.12, and includes the gene encoding the four-transmembrane protein MAL2 [9], which is increased in copy number and/or overexpressed in breast and other cancers [10-18]. MAL2 is a 176 amino acid protein that contains a MARVEL (MAL and related proteins for vesicle trafficking and membrane link) domain commonly identified in proteins associated with membrane apposition events [19], and is an essential component of the basolateral-to-apical transcytotic machinery [20]. Increased MAL2 expression in ovarian cancer has been repeatedly identified by independent expression microarray studies [21-24], with two meta-analyses highlighting the same finding [22,25]. Increased *MAL2 *expression has been validated in other cancer types using RT-PCR [26,27], and demonstrated at the protein level in renal cell [18,28] and breast carcinomas [12]. However, no study to date has examined whether MAL2 expression is increased in ovarian carcinoma, or its potential clinical significance.

MAL2 is known to bind the product of another gene on chromosome 8q, tumor protein D52 (TPD52) [9,29], which is a member of the similarly-named gene and protein family [30]. The *TPD52 *gene maps to chromosome 8q21.13, and demonstrates copy number increases and overexpression in a variety of cancers [reviewed in 31]. The TPD52 protein is 184 amino acids in length and contains a coiled-coil domain, but does not show significant levels of sequence identity to proteins beyond the TPD52-like family [30]. Its expression in normal secretory epithelia has been implicated in regulating exocytotic secretion [32], whereas exogenous TPD52 expression in cultured cell lines results in increased proliferation and anchorage-independent growth [12,33,34], and in vivo metastasis in immunocompetent hosts [34]. In ovarian cancer, TPD52 overexpression has been identified in all histological subtypes of ovarian carcinoma relative to normal ovarian epithelium, with a significant positive correlation between TPD52 expression and gene copy number being found in an independent serous carcinoma cohort [3]. Other studies have similarly reported increased TPD52 expression in ovarian cancer using expression microarray [23,24,35,36] and proteomic approaches [37]. While high TPD52 expression in breast cancer has been reported to be an adverse prognostic factor [12], the clinical significance of increased TPD52 expression in ovarian cancer has not been directly investigated.

The aim of the present study was therefore to define MAL2 and TPD52 expression in a large cohort of ovarian carcinomas, relative to other clinical parameters. Immunohistochemical staining using previously described polyclonal antisera [3,12,29] was assessed both visually and digitally, as previously described in breast carcinoma [12].

## Methods

### Tissue and clinicopathological data

The patient cohort (n = 289) were women undergoing primary laparatomy at the Gynaecological Cancer Centre, Royal Hospital for Women, Sydney, between 1989 and 2002. Formalin-fixed, paraffin-embedded tissue specimens were collected retrospectively and surgical, clinical and histopathological data (histopathological diagnosis, FIGO stage, surgical debulking, tumour grade, survival) were extracted from medical records. All experimental procedures were approved by the Human Research Ethics Committee of the Sydney South East Area Hospital Service, Northern Section (00/115).

### Immunohistochemical analysis of paraffin-embedded ovarian tissue microarrays

Construction of the tissue microarrays used in this study has been previously described [22]. Immunohistochemical staining was performed using a DAKO autostainer (DAKO, Glostrup, Denmark). Tissue sections were dewaxed and rehydrated according to standard protocols, followed by antigen retrieval in a 100°C water bath (MAL2: 0.5 × Target Retrieval Solution pH 6 (DAKO) for 20 min; TPD52: 1 × Target Retrieval Solution pH 9 (DAKO) for 1 h). The TPD52 and MAL2 antisera employed for immunohistochemistry have both been previously described [3,12,29]. Slides were incubated for 1 h with affinity-purified TPD52 (1/50) or MAL2 (1/100) antibodies. Primary antibody was omitted in control incubations. Bound antibody was detected by LINK/EnVision using 3,3'-diaminobenzidine Plus (DAKO) as substrate. Counterstaining was performed with hematoxylin and 1% acid alcohol.

Scoring was assessed by two gynaecological pathologists (R.M. and J.P.S) blinded to patient outcome, and discrepancies resolved by discussions around a multi-head microscope. Immunohistochemical staining intensity was scored as 0 (absent), 1 (low), 2 (moderate) and 3 (high), and immunohistochemical staining extent was scored as a percentage of the relevant tissue core compartment. Staining intensity and extent values were subsequently multiplied to produce histoscores (possible range 0 (0 × 0%) to 300 (3 × 100%)). Slides were also independently digitally scanned using a Virtual Microscope ScanScope Unit and ScanScope Console program at 200 × magnification, and visualised using Image Scope (Aperio Technologies, Vista, CA). Staining intensity and extent were quantified within tissue cores of fixed and uniform diameter using the Positive Pixel Count algorithm (Aperio Technologies), with partial tissue cores, those with staining artefacts or without epithelial elements (normal or cancerous) being excluded. The strong pixel count (SPC), defined as the number of pixels of 175-220 intensity, was measured per tissue core, and SPC values for replicate cores were averaged.

### Statistical analyses

The SPSS for Windows package (Version 17, SPSS Inc., Chicago, IL) was used in all analyses. Distributions of continuous variables were often skewed, and summarised using medians and interquartile ranges. Categorical variables were summarised using percentages within each group, with differences in proportions between groups being compared using Fisher's Exact Test. The Mann-Whitney U test was used to test for differences in MAL2 and TPD52 SPCs or histoscores between sample groups. Spearman's rank correlation was used to compare protein expression and other parameters. Survival distributions were estimated by the Kaplan-Meier method, and the significance of differences between overall survival rates was ascertained using the log-rank test. Multiple Cox proportional hazards models with backward step-wise selection were used to identify independent predictors of survival from potential risk factors. Length of survival was defined from the date of initial diagnosis to the date of patient death or in the case of surviving patients, their most recent follow-up date. Patients who were alive at their most recent follow-up or lost to further follow-up were censored.

## Results

MAL2 and TPD52 expression were assessed in ovarian tissue samples (Table 1) using both visual scoring by experienced pathologists, and digital scoring to provide independent quantitation of immunohistochemical staining. In order to identify robust findings, we paid particular attention to statistically different staining levels obtained from comparisons of both visually-scored staining, and independently derived SPCs (Tables 2, 3). We considered visually-scored staining intensity values both alone, and in combination with staining extent as calculated histoscores, but found that the latter combination did not produce additional insights beyond those obtained through comparing visual staining intensities alone (Tables 2, 3).

**Table 1:** Patient cohort

Clinical Variable	All	High-Grade Serous^a^	Clear Cell	Endometrioid	Mucinous	Low-Grade Serous^b^
No. cases (% of total)	289 (100%)	176 (61%)	8 (3%)	22 (8%)	49 (17%)	34 (12%)

Median Age (years)	58.8	60.4	56.1	54.3	57.5	46.4

Benign	7	0	0	0	7	0

Borderline	61	0	0	22	32	27

Stage I^c^	79	11	6	14	35	13

Stage II	16	7	0	4	1	4

Stage III	155	131	2	1	5	16

Stage IV	30	27	0	2	0	1

Grade 1^d^	22	0	N/A	8	7	7

Grade 2	85	78	N/A	5	2	0

Grade 3	106	98	N/A	7	1	0

Residual Disease ≤ 1 cm^e^	151	84	1	9	34	23

Residual Disease >1 cm	137	91	7	13	15	11

Deceased	146	126	2	5	9	4

^a^Includes one tumour of mixed histology of which serous was the predominant type.

^b^Borderline serous tumours are included in the low-grade serous column.

^c^Staging information missing for 2 patients (no staging for benign tumours).

^d^Clear cell carcinomas were not graded.

^e^Residual disease information missing for 1 patient.

**Table 2:** Statistical comparisons of MAL2 immunohistochemical staining in ovarian tissue samples

		Visual scoring		Automated scoring^b^	
Tissue samples compared		Intensity^a^	Histoscores^b^		
Serous histology	Low-grade	2/7 (29%) v. 0/27 (0%)	100 (10-240) v. 86 (65-146)	422 (38-1,177) v. 235 (137-416)	
	carcinoma v.	p = 0.037, n = 34	NS^c^, n = 34	NS, n = 34	
	borderline				
	
	**High-grade**	**100/175 (57%) v. 0/27 (0%)**	**255 (173-291) v. 86 (65-146)**	**480 (187-846) v. 235 (137-416)**	
	**carcinoma v**.	**p < 0.001, n = 202**^d^	**p < 0.001, n = 202**	**p = 0.003, n = 192**	
	**borderline**				

Mucinous histology	Carcinoma v.	5/10 (50%) v. 3/32 (9%)	127 (0-296) v. 30 (0-79)	57 (18-768) v. 47 (27-132)	
	borderline	p = 0.012, n = 42	NS, n = 42	NS, n = 42	
	
	Carcinoma v.	5/10 (50%) v. 0/7 (0%)	127 (0-296) v. 0 (0-80)	57 (18-768) v. 224 (71-301)	
	benign	p = 0.044, n = 17	NS, n = 17	NS, n = 17	

Carcinomas	**Serous v**.	**102/182 (56%) v. 6/20 (30%)**	**198 (82-285) v. 93 (0-237)**	**528 (166-1,060) v. 72 (16-267)**	
	**endometrioid**	**p = 0.033, n = 202**	**p = 0.019, n = 202**	**p < 0.001, n = 192**	
	
	Serous v.	102/182 (56%) v. 5/10 (50%)	198 (82-285) v. 127 (0-296)	528 (166-1,060) v. 57 (18-768)	
	mucinous	NS, n = 192	NS, n = 192	p = 0.030, n = 182	
	
	Clear cell v.	3/8 (38%) v. 6/20 (30%)	113 (40-249) v. 93 (0-237)	231 (133-661) v. 72 (16-267)	
	endometrioid	NS, n = 28	NS, n = 28	NS, n = 28	
	
	Clear cell v.	3/8 (38%) v. 102/182 (56%)	113 (40-249) v.198 (82-285)	231 (133-661) v. 528 (166-1,060)	
	serous	NS, n = 190	NS, n = 190	NS, n = 180	
	
	**High-grade**	**100/175 (57%) v. 16/46 (35%)**	**200 (96-285) v. 110 (8-244)**	**531 (166-1,059) v. 124 (20-415)**	
	**serous v. others**	**p = 0.008, n = 221**	**p = 0.002, n = 221**	**p < 0.001, n = 211**	

^a^Comparisons of proportions (percentages) of samples with high level immunohistochemical staining, Fisher's Exact Test.

^b^Comparisons of median histoscores or strong pixel counts (SPCs, shown in thousands), Mann-Whitney Test. Median values (interquartile ranges) are shown.

^c^Not statistically significant at p < 0.05.

^d^Values in bold indicate associations that were significant when visual scores and digitally-determined SPCs were compared.

**Table 3:** Statistical comparisons of TPD52 immunohistochemical staining in ovarian tissue samples

Tissue samples compared		Visual scoring		Automated scoring	
		Intensity^a^	Histoscores^b^	SPCs^b^	
Serous histology	Low-grade	4/5 (80%) v. 11/24 (46%)	182 (155-248) v. 181 (163-272)	260 (223-871) v. 237 (90-359)	
	carcinoma v.	NS, n = 29	NS, n = 29	NS, n = 34	
	borderline				
	
	**High-grade**	**111/162 (69%) v. 11/24 (46%)**	**200 (96-285) v. 181 (163-272)**	**530 (166-1,059) v. 237 (90-359)**	
	**carcinoma v**.	**p = 0.038, n = 186**	**p = 0.038, n = 186**	**p < 0.001, n = 192**	
	**borderline**				

Mucinous histology	Carcinoma v.	8/10 (80%) v. 18/31 (58%)	236 (140-296) v. 216 (100-285)	384 (68-729) v. 125 (33-235)	
	borderline	NS, n = 41	NS, n = 41	NS, n = 29	
	
	Carcinoma v.	8/10 (80%) v. 2/6 (33%)	236 (140-296) v. 195 (140-225)	384 (68-729) v. 63 (8-131)	
	benign	NS, n = 16	NS, n = 16	p = 0.032, n = 17	

Carcinomas	Serous v.	115/167 (69%) v. 9/19 (47%)	255 (171-291) v. 180 (90-285)	479 (190-848) v. 116 (22-337)	
	endometrioid	NS, n = 186	NS, n = 186	p = 0.003, n = 192	
	
	Serous v.	115/167 (69%) v. 8/10 (80%)	255 (171-291) v. 236 (140-296)	479 (190-848) v. 383 (69-729)	
	mucinous	NS, n = 177	NS, n = 177	NS, n = 182	
	
	**Clear cell v**.	**8/8 (100%) v. 9/19 (47%)**	**288 (283-294) v. 180 (90-285)**	**534 (287-1,061) v. 116 (22-337)**	
	**endometrioid**	**p = 0.012, n = 27**	**p = 0.019, n = 27**	**p = 0.019, n = 28**	
	
	Clear cell v.	8/8 (100%) v. 115/167 (69%)	288 (283-294) v. 255 (171-291)	534 (287-1,061) v. 479 (190-848)	
	serous	NS, n = 175	p = 0.028, n = 175	NS, n = 180	
	
	High-grade serous	111/162 (69%) v. 30/43 (70%)	255 (173-291) v. 264 (165-291)	480 (187-846) v. 274 (84-715)	
	v. others	NS, n = 205	NS, n = 205	p = 0.048, n = 211	

^a^Comparisons of proportions (percentages) of samples with high level immunohistochemical staining, Fisher's Exact Test.

^b^Comparisons of median histoscores or strong pixel counts (SPCs, shown in thousands), Mann-Whitney Test. Compared median values (interquartile ranges) are shown.

^c^Not statistically significant at p < 0.05.

^d^Values in bold indicate associations that were significant when visual scores and digitally-determined SPCs were compared.

**Figure 1:**
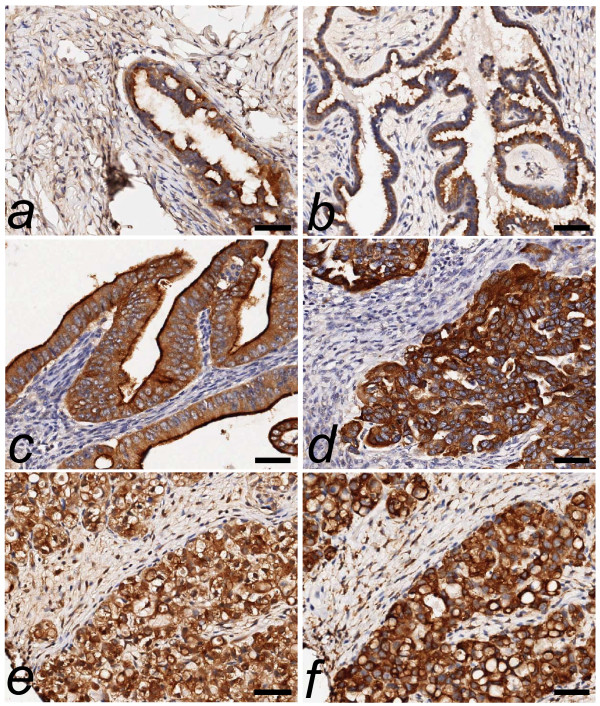
**Immunohistochemical detection of MAL2 or TPD52 (brown staining) within paraffin-embedded ovarian tissue sections counterstained with hematoxylin**. MAL2 staining within a (**a**) benign serous lesion; (**b**) serous borderline lesion; (**c**) mucinous borderline lesion; (**d**) serous carcinoma; (**e**) clear cell carcinoma; and (**f**) TPD52 staining in the same clear cell carcinoma shown in (**e**) for comparison. Scale bar = 50 μm.

As predicted from previous analyses of MAL2 intracellular localisation [12,18,28], MAL2 immunohistochemical staining displayed cytoplasmic and/or membrane sub-cellular localisations in the samples examined (Fig. 1). MAL2 cytoplasmic and membrane staining intensities were significantly positively correlated in the tumour cohort (Spearman's rank correlation coefficient, r_s _= 0.736, p < 0.001, n = 207), and both were significantly positively correlated with measured SPCs in tumour cores (r_s _= 0.599, p < 0.001, n = 211 for cytoplasmic staining; r_s _= 0.592, p < 0.001, n = 197 for membrane staining). As cytoplasmic staining was indicated to contribute more significantly to SPCs than membrane staining (see below), analyses of visually-scored MAL2 staining intensity focussed upon cytoplasmic staining, unless otherwise indicated.

**Figure 2:**
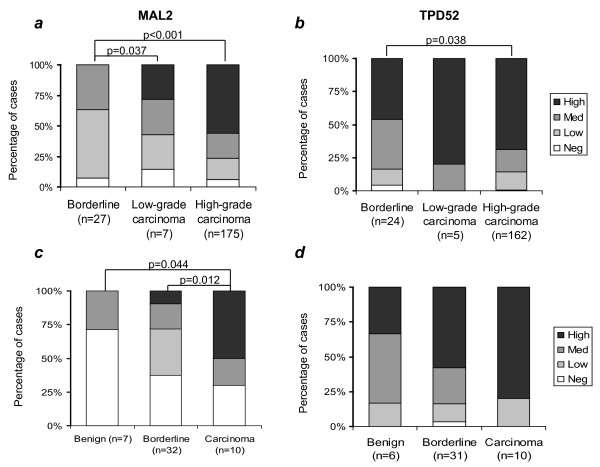
**Graphical representations of visual scores of MAL2 (a, c) or TPD52 (b, d) staining intensity in serous borderline tumours and low-grade (grade 1) or high-grade (grade 2 or 3) serous carcinomas (a, b) and in mucinous cystadenomas, borderline tumours and carcinomas (c, d)**. Information concerning the FIGO stages and histological grades of tumours analysed is shown in Table 1. Sample numbers in each category are indicated below X-axes. Significant differences between the proportions of cases with high-level immunohistochemical staining (p values, Fisher's Exact Test) are indicated.

Different tumourigenic pathways have been proposed in the development of borderline and low-grade serous carcinomas (Type I) versus high-grade serous carcinomas (Type II) [2]. MAL2 and TPD52 staining was therefore compared in these groups. In the case of both proteins, more low-grade serous carcinoma (grade 1) showed high-level cytoplasmic staining compared with serous borderline tumours (Fig. 2a, b), however this only reached statistical significance for visual scoring of MAL2 staining (Tables 2, 3). Significantly greater proportions of high-grade serous carcinomas (grades 2 and 3) showed high-level cytoplasmic staining of both MAL2 and TPD52 compared with serous borderline tumours, and this was supported by SPCs which were significantly increased in high-grade serous carcinomas compared with serous borderline tumours (Tables 2, 3). High-level MAL2 staining at the membrane was frequent in serous borderline lesions (17/27, 63%), but this did not appear to significantly contribute to measured SPCs (data not shown). Neither MAL2 nor TPD52 was differentially expressed in serous carcinomas according to FIGO stage or histological grade, and no significant correlations were measured between either FIGO stage or grade and SPCs or staining intensity (data not shown). Comparisons of MAL2 and TPD52 expression were also made in a smaller cohort of mucinous carcinomas, borderline tumours and cystadenomas (Fig. 2c, d, Tables 2, 3). While high-level MAL2 and TPD52 staining were both frequent in mucinous carcinoma (Fig. 2c, d), and apparently increased stepwise from benign to borderline to carcinoma, this was not confirmed by comparisons of both visually and digitally-scored staining values (Tables 2, 3).

**Figure 3:**
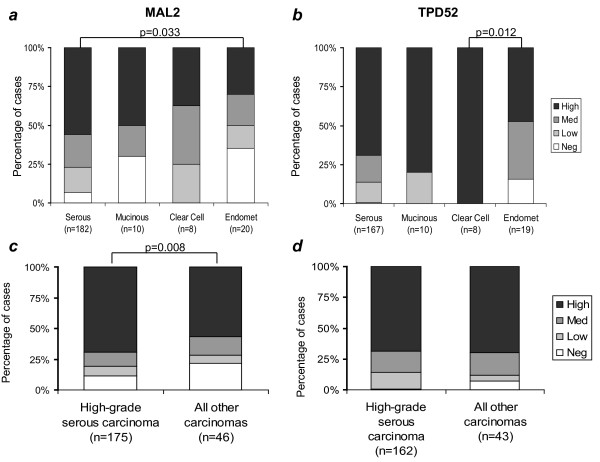
**Graphical representations comparing visual scores of MAL2 (a, c) and TPD52 (b, d) staining intensity in ovarian carcinomas according to histological subtype**. Information concerning the FIGO stages and histological grades of tumours analysed is shown in Table 1. Sample numbers in each category are indicated below the X-axes. Significant differences between either the proportions of cases with high-level immunohistochemical staining (p values, Fisher's Exact Test) are indicated.

MAL2 and TPD52 staining were also compared in carcinomas according to histological subtype, and here differences between MAL2 and TPD52 expression emerged (Fig. 3). High-level MAL2 staining was most frequent in serous carcinomas (102/182, 56%), followed by mucinous (5/10, 50%), clear cell (3/8, 38%) and endometrioid subtypes (6/20, 30%) (Fig. 3a), and serous carcinomas also displayed the highest median MAL2 SPC (Table 2). High-level MAL2 staining was significantly more frequent and MAL2 SPCs were significantly higher in serous than endometrioid carcinomas (Fig. 3a, Table 2). In contrast, high-level TPD52 staining was most frequent in clear cell carcinomas (8/8, 100%), followed by mucinous (8/10, 80%) serous (115/167, 69%), and endometrioid carcinomas (9/19, 47%) (Fig. 3b), with clear cell carcinomas also displaying the highest median TPD52 SPC (Table 3). High-level TPD52 staining was more frequent and TPD52 SPCs were significantly elevated in clear cell carcinomas relative to endometrioid carcinomas (Fig. 3b, Table 3). As MAL2 was most frequently overexpressed in serous carcinomas, we compared MAL2 and TPD52 staining in high-grade serous carcinomas versus all others (Fig. 3c, d, Tables 2, 3). Statistical comparisons reproducibly highlighted that MAL2 staining was higher in high-grade serous carcinomas, whereas TPD52 was indicated to be more equivalently expressed (Fig. 3c, d, Tables 2, 3).

**Figure 4:**
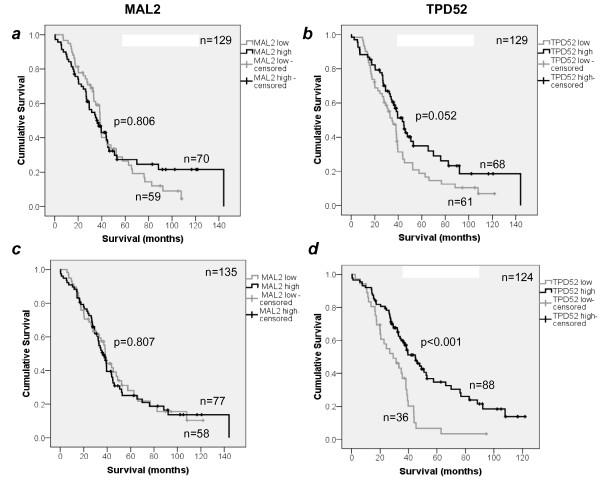
**Kaplan-Meier plots comparing overall patient survival (X axis, in months) according to MAL2 (a, c) or TPD52 (b, d) expression status in stage III serous ovarian carcinomas**. In panels **a **and **b**, patient cohorts are dichotomised at median MAL2 (**a**) or TPD52 (**b**) SPC values measured in the carcinoma cohort. In panels **c **and **d**, tumours with high-level cytoplasmic staining for MAL2 (**c**) or TPD52 (**d**), as determined by visual scoring, are compared with all others. In all panels, high-expressing cases are shown in black, whereas low-expressing cases are shown in grey. Numbers of patients compared in each arm, and associated p values (log rank tests) are indicated.

To examine the clinical significance of MAL2 and TPD52 overexpression, survival analyses were carried out considering MAL2 and TPD52 staining as both visually and digitally scored data (Fig. 4). Given that significant differences in survival between histological subtypes and stages of ovarian cancer, initial analyses were confined to stage III serous carcinoma. The inclusion or exclusion of grade 1 cancers within this cohort did not significantly affect the statistical results obtained (data not shown). Dividing the stage III serous carcinoma cohort according to median MAL2 or TPD52 SPC tumour values indicated similar overall survival according to MAL2 staining (Fig. 4a), but a trend towards improved overall survival with increased TPD52 staining (Fig. 4b). Similarly, comparable overall survival was noted for tumours with high visually-scored MAL2 levels relative to all others (Fig. 4c), whereas significantly improved overall survival was noted in patients with tumours with high TPD52 staining (log-rank test, p < 0.001, n = 124) (Fig. 4d). Multivariate analyses identified high TPD52 staining as an independent predictor of survival, both in the overall carcinoma cohort (hazard ratio (HR), 0.498; 95% confidence interval (CI), 0.340-0.728; p < 0.001; n = 221), and in serous carcinomas only (HR, 0.440; 95% CI, 0.294-0.658; p < 0.001; n = 182), after adjustment for age at diagnosis, FIGO stage, histological grade, and presence of residual disease (nil or < 1 cm versus >1 cm). Similar results were also obtained when cohorts were divided around median TPD52 SPC values (overall cohort: HR, 0.657; 95% CI, 0.461-0.937; p = 0.020; n = 221; serous cohort: HR, 0.637; 95% CI, 0.442-0.918; p = 0.015; n = 182). These analyses indicate that high TPD52 expression is a favourable independent prognostic factor in ovarian carcinoma, whereas no significant associations between MAL2 expression and overall patient survival were detected.

## Discussion

The present study has confirmed MAL2 overexpression in ovarian cancer, as predicted by previous expression microarray analyses [21-25], and has shown this to be a frequent event. MAL2 was significantly overexpressed in high-grade serous carcinomas compared with borderline tumours, and in high-grade serous carcinomas relative to the remaining histological subtypes combined. High-level MAL2 expression was also noted in mucinous, clear cell and endometrioid ovarian carcinomas, albeit less frequently than in serous carcinomas. The frequent overexpression of MAL2 in ovarian carcinoma, coupled with its low expression in benign and borderline lesions, may indicate a potential role for MAL2 in disease detection and/or monitoring, particularly in high-grade serous carcinoma. This is consistent with the previous inclusion of MAL2 in marker panels to discriminate pancreatic cancer from pancreatitis [26] and metastatic from non-affected lymph nodes in colorectal cancer patients [27].

Frequent reports of MAL2 overexpression in numerous cancer types, in association with increases in gene copy number, argue against MAL2 overexpression being an epiphenomenon. MAL2 has been recently shown to bind the MUC1 oncoprotein [29], and *MAL2 *transcripts are predicted to be co-expressed with *EpCAM *[38]. However, it is not yet clear how MAL2 overexpression promotes tumourigenesis or progression. Increased Myc-tagged MAL2 expression in MCF-10A breast epithelial cells produced reduced proliferation rates and an elongated cell phenotype [29]. Furthermore, other studies examining gene expression consequences of oncogene overexpression have reported reduced *MAL2 *expression in these models [39,40]. MAL2 overexpression may therefore contribute to tumourigenesis through mechanisms that do not emerge from the study of homogenous *in vitro *systems. These could include modulating cell-cell interactions or signalling between cancer and/or other cell types. It was interesting to note that cytoplasmic expression of MAL2 was rare in benign and borderline tumours, but common in ovarian carcinomas. MAL2 also showed a broad cytoplasmic distribution in renal cell carcinomas [18,28] and in breast carcinomas and cell lines [12,29]. This might indicate that MAL2 plays an oncogenic function in some cell types when distributed throughout the cytoplasm.

Like MAL2, TPD52 was overexpressed in high-grade serous carcinomas relative to borderline tumours, but was more equivalently expressed in high-grade serous tumours relative to the combined cohort of other histological subtypes. This agrees with our previous finding that TPD52 was broadly overexpressed in an independent ovarian carcinoma cohort [3]. The present study identified high TPD52 staining as particularly characteristic of clear cell carcinomas, as this was detected in all cases examined. Frequent high-level TPD52 expression in clear cell carcinoma is consistent with the results of array-based comparative genomic hybridisation analyses of ovarian clear cell carcinoma cell lines, where increased *TPD52 *copy number was noted in 6/12 cell lines examined [8].

Despite MAL2 overexpression being more frequent in high-grade serous carcinoma, MAL2 expression was not significantly associated with overall patient survival in this study. In contrast, increased TPD52 staining was noted to be a favourable prognostic marker in ovarian carcinoma. While this finding derives from the analysis of immunohistochemical staining as a single technique, a recent expression microarray study also identified *TPD52 *overexpression as being associated with improved progression-free and overall survival in patients with serous and endometrioid tumours [24]. It may also be relevant that chromosome 8q21 gain has been previously associated with improved survival in clear cell carcinoma patients [41]. However, the finding that TPD52 is a favourable prognostic indicator in ovarian carcinoma patients contrasts with findings obtained in breast cancer [12]. Here, high TPD52 expression was an unfavourable indicator, both within the cohort overall, and patient subgroups already characterised by poorer survival [12]. This is also consistent with reports of TPD52 being included in gene signatures associated with unfavourable prognosis [42,43], and with the clinical significance of chromosome 8q21 gain in breast cancer [44-47]. Thus, whereas increased TPD52 expression appears to be a common event in both breast and ovarian carcinoma, the contrasting clinical significance of both TPD52 overexpression and chromosome 8q21 gain suggests different roles for TPD52 in these cancer types. There is evidence that TPD52 may promote invasion through solid tissues, as suggested by the finding that Tpd52-expressing 3T3 cells injected into the mammary fat pad subsequently formed lung metastases in immunocompetent hosts [34]. In contrast, the pattern of metastatic spread is very different in ovarian carcinoma, as there is no anatomical barrier to widespread tumour dissemination and spread within the peritoneal cavity [1]. Such differences in tumour dissemination patterns could partially explain the opposing prognostic significance of increased TPD52 expression or copy number reported in breast [12,42-44] and ovarian cancer [24]. Alternatively, a comparative lack of TPD52 expression could segregate with adverse prognostic markers in ovarian carcinoma, and thus the association between increased TPD52 expression and improved overall survival may be indirect.

## Conclusion

The present study has highlighted frequent MAL2 overexpression in ovarian carcinoma, particularly in serous tumours. The frequent overexpression of MAL2 in ovarian carcinomas, coupled with its low expression in benign and borderline lesions, suggest that MAL2 may be a useful marker component to assist in disease detection and/or monitoring. High TPD52 staining was associated with significantly improved overall patient survival in ovarian carcinoma. The differential association of high TPD52 staining and overall patient survival in breast versus ovarian carcinoma may indicate different roles for TPD52 overexpression in ovarian versus breast tumour progression.

## Abbreviations

TPD52: tumor protein D52; MARVEL: MAL and related proteins for vesicle trafficking and membrane link; RT-PCR: reverse transcription-polymerase chain reaction; SPC: strong pixel count; FIGO: International Federation of Gynaecology and Obstetrics; NS: not statistically significant; HR: hazard ration; CI: confidence interval.

## Competing interests

The authors declare that they have no competing interests.

## Authors' contributions

PMOB conceived of this study. JAB and PMOB contributed to study design and co-ordination, carried out the statistical analyses, and drafted the manuscript. SM, JRH, BSG, SF and CE carried out immunohistochemical staining experiments and/or contributed to data analyses. RM and JPS conducted the pathological review of all samples and immunohistochemical staining. NFH, RLS, and AdF contributed to study design and co-ordination, and helped draft the manuscript. All authors have read and approved the final manuscript.

## Pre-publication history

The pre-publication history for this paper can be accessed here:

http://www.biomedcentral.com/1471-2407/10/497/prepub
